# Factors structuring microbial communities in highly impacted coastal marine sediments (Mar Menor lagoon, SE Spain)

**DOI:** 10.3389/fmicb.2022.937683

**Published:** 2022-09-07

**Authors:** Borja Aldeguer-Riquelme, Esther Rubio-Portillo, José Álvarez-Rogel, Francisca Giménez-Casalduero, Xose Luis Otero, María-Dolores Belando, Jaime Bernardeau-Esteller, Rocío García-Muñoz, Aitor Forcada, Juan M. Ruiz, Fernando Santos, Josefa Antón

**Affiliations:** ^1^Department of Physiology, Genetics, and Microbiology, University of Alicante, Alicante, Spain; ^2^Department of Agricultural Engineering of the Escuela Técnica Superior Ingeniería Agronómica (ETSIA) & Soil Ecology and Biotechnology Unit of the Institute of Plant Biotechnology, Technical University of Cartagena, Cartagena, Spain; ^3^Department of Marine Science and Applied Biology, University of Alicante, Alicante, Spain; ^4^Cross-Research in Environmental Technologies (CRETUS), Departamento de Edafoloxía e Química Agrícola, Facultade de Bioloxía, Universidade de Santiago de Compostela, Santiago de Compostela, Spain; ^5^Seagrass Ecology Group, Spanish Oceanography Institute of the Spanish National Research Council, Oceanography Center of Murcia, Murcia, Spain; ^6^Multidisciplinary Institute of Environmental Studies Ramón Margalef, University of Alicante, Alicante, Spain

**Keywords:** 16S rRNA gene amplicon sequencing, coastal lagoon, Mar Menor, sediment, microbial community

## Abstract

Coastal marine lagoons are environments highly vulnerable to anthropogenic pressures such as agriculture nutrient loading or runoff from metalliferous mining. Sediment microorganisms, which are key components in the biogeochemical cycles, can help attenuate these impacts by accumulating nutrients and pollutants. The Mar Menor, located in the southeast of Spain, is an example of a coastal lagoon strongly altered by anthropic pressures, but the microbial community inhabiting its sediments remains unknown. Here, we describe the sediment prokaryotic communities along a wide range of environmental conditions in the lagoon, revealing that microbial communities were highly heterogeneous among stations, although a core microbiome was detected. The microbiota was dominated by *Delta*- and *Gammaproteobacteria* and members of the *Bacteroidia* class. Additionally, several uncultured groups such as *Asgardarchaeota* were detected in relatively high proportions. Sediment texture, the presence of *Caulerpa* or *Cymodocea*, depth, and geographic location were among the most important factors structuring microbial assemblages. Furthermore, microbial communities in the stations with the highest concentrations of potentially toxic elements (Fe, Pb, As, Zn, and Cd) were less stable than those in the non-contaminated stations. This finding suggests that bacteria colonizing heavily contaminated stations are specialists sensitive to change.

## Introduction

Coastal lagoons are highly biodiverse and productive environments that provide critical ecosystem services with significant socioeconomic impacts such as food provision or climate regulation (Newton et al., 2018). They are shallow water bodies separated from the ocean by a barrier and connected to it by one or more restricted inlets (Kjerfve, 1994). Due to their proximity to land, small volumes, and long water renewal time, coastal lagoons are extremely sensitive to anthropic eutrophication and pollutant accumulation, such as agricultural nutrient loading, fishing, tourism, and mining (Barbier et al., 2011; Pérez-Ruzafa and Marcos, 2012), which may promote sudden and drastic changes in ecosystem structure, functions, and services (De Wit et al., 2001).

Sediments are a key compartment for the functioning and quality of coastal lagoons by acting as a sink for organic matter (Hewson and Fuhrman, 2008), potentially toxic elements (PTEs) (i.e., metals and metalloids), and inorganic nutrients such as N or P (Jiménez-Cárceles et al., 2006; Sharifuzzaman et al., 2016). Conversely, sediments can transfer nutrients and PTEs into the water column by diffusion or when sediment particles are suspended by winds and currents. Due to the presence of a high nutrient load in sediments, resident microbial communities are usually very dense, reaching up to 10^9^ cells/g (Luna et al., 2002; Petro et al., 2019), which is 3-4 orders of magnitude higher than microbial densities in the water column (DeLong et al., 1999; Eilers et al., 2000). Furthermore, diffusion of O_2_ through the sediment is limited (Hewson and Fuhrman, 2008), which leads to redox zoning and changes in microbial metabolism. At the surface, organic matter is oxidized by aerobic microbes that deplete available O_2_; in deeper layers, anaerobes use alternative electron acceptors such as NO_3_^–^, MnO_2_, amorphous FeOOH, and SO_4_^2–^ (Sørensen et al., 1979). Due to their high abundances and metabolic diversity, microorganisms in marine sediments are a key factor in the remineralization of nutrients (Alongi, 1994, 1998); and thus, they have a strong influence on the biogeochemical cycles of coastal marine ecosystems.

**FIGURE 1:**
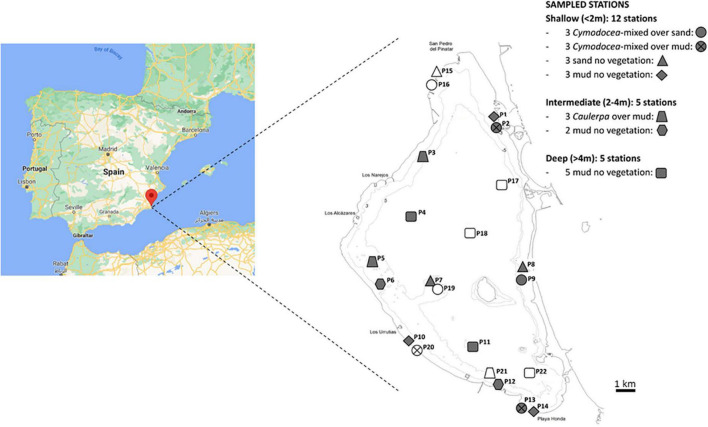
Geographic location and sampling stations in the Mar Menor lagoon. Levels of different factors (depth, vegetation, and texture) are indicated by symbols (see legend at the right) for each sampling site. The symbols colored in gray indicate stations selected for microbiological studies.

The Mar Menor, located in the southeast of Spain (Figure 1), is the largest hypersaline (i.e., 42–47 psu) coastal lagoon in Europe and provides a paradigmatic example of a coastal marine ecosystem that is highly valuable yet strongly altered by several anthropogenic factors (Marín-Guirao et al., 2005; Conesa and Jiménez-Cárceles, 2007; León et al., 2013, 2017, 2018; Jiménez-Martinez et al., 2016; Romero et al., 2020). In the 1970s, one inlet (El Estacio channel) connecting the lagoon with the Mediterranean Sea was broadened (Mas, 1994), drastically changing the hydrodynamic regime and decreasing the lagoon temperature and salinity toward the values closer to those typically measured in the adjacent Mediterranean Sea (37 psu). This event, together with the massive loading of urban sewage into the lagoon, allowed the spread of the opportunistic chlorophyte *Caulerpa prolifera* (Ballester, 1985; Terrados and Ros, 1992). Furthermore, due to the past extractive activity in the Cartagena-La Unión mining district, located close to the southern basin (Conesa and Schulin, 2010), PTEs such as Pb, Zn, Cd, and As have been loaded into the lagoon by hydric and aeolian erosion and surface and subsurface runoffs. These PTEs accumulate in the sediment (Simonneau, 1973; García and Muñoz-Vera, 2015) or are taken up by vegetation and fauna (Serrano et al., 2019; Romero et al., 2020). In addition, the increase in tourism activity since the 1960s, and the effects of intensive agriculture since the 1970s, have deeply transformed the surrounding territory and poured huge amounts of sediments, nutrients, and contaminants into the lagoon (Jiménez-Martinez et al., 2016; Pérez-Ruzafa et al., 2019).

These impacts have shifted the lagoon from an oligotrophic system dominated by the seagrass *Cymodocea nodosa* (Ballester, 1985), to an eutrophic system that caused the macroalga *C. prolifera* to rapidly colonize, forming monospecific or mixed meadows with *C. nodosa* (Ballester, 1985; Terrados and Ros, 1991) (hereafter we will refer to these species as *Caulerpa* and *Cymodocea*, respectively). Contaminants of emerging concern, such as pesticides, polycyclic aromatic hydrocarbons (PAHs), surfactants, and pharmaceuticals, have also been detected in the lagoon-associated watercourses, water columns, sediments, and even in bivalves and fishes (Moreno-González et al., 2014, 2016; Jiménez-Martinez et al., 2016). Furthermore, the lagoon experienced a unique and severe event of the so-called “ecosystem disruptive algal bloom” as a consequence of a phytoplankton overgrowth initiated in 2016 by the cyanobacterium *Synechoccocus* (Aguilar-Escribano et al., 2016; Mercado et al., 2021). This caused the water turbidity to increase up to critical levels for macrophyte survival and led to the sudden loss of 82% of benthic vegetation, including *Caulerpa* and *Cymodocea* (Belando-Torrente et al., 2019; Marín-Guirao et al., 2019).

Some studies have addressed the effects of these anthropic impacts on the macro-organisms inhabiting the lagoon (Muñoz-Vera et al., 2015; Serrano et al., 2019; Romero et al., 2020) and on benthic food webs (Marín-Guirao et al., 2008). However, the information available on the microbial ecology of the system is very scarce and restricted to the water column (Gilabert, 2001; Ghai et al., 2012). Thus, despite the ecological relevance of sediment microbiota in coastal lagoons, to date, nothing is known about this component of the microbial assemblage in the Mar Menor. To fill this gap, we aimed at ascertaining whether (i) the taxonomic composition and inferred putative metabolic capabilities of the Mar Menor sediments were similar to that of other marine sediments, (ii) factors such as texture, depth, vegetation, and geographical location could be linked to the microbial community structure, and (iii) microbial communities in each station underwent temporal changes.

Our results showed that the microbial communities in the analyzed sediments were highly heterogeneous among stations, although a core microbiome could be detected. Sediment texture, vegetation type, depth, and geographical location were found to drive the structure of the microbial community, while time was only relevant in the most PTE contaminated stations. Mining activity-derived PTEs, which were present in extremely high concentrations in the south of the lagoon, also affected prokaryotic composition and stability, indicating the capacity of sustained human pressures to alter sediment microbiomes and, likely, their ecosystem functions.

## Materials and methods

### Study area

The Mar Menor is a hypersaline shallow coastal lagoon (maximum depth 7 m and average depth 4.5 m) located in the Region of Murcia, southeast of Spain, with an area of 135 km^2^. It is separated from the Mediterranean Sea by a sand bar of 22 km length (Figure 1) and connected to it by three small inlets through which water exchanges between both water bodies occur. The Mar Menor water mass is then relatively confined with a renewal time of about 1 year (Fraile-Nuez et al., 2018) and has a salinity range of 42–47 psu (Aravio-Torre and Arévalo, 1973), higher than that of the adjacent Mediterranean Sea in this area (37–38.5 psu; Pérez-Ruzafa et al., 2004). Water temperature varies from 10°C in winter to 31°C in summer, without a significant stratification in the water column (Fraile-Nuez et al., 2018). Nitrate concentrations were below 1 μM until the 1990s when agricultural and urban wastes increased the concentration above 8 μM (Ruiz et al., 2020). Curiously, chlorophyll concentrations did not significantly increase above 1–3 mg/m^3^ until 2016, when the macrophyte populations in the Mar Menor lagoon collapsed due to a massive phytoplankton bloom (Belando-Torrente et al., 2019; Pérez-Ruzafa et al., 2019; Ruiz et al., 2020).

### Field sampling

A total of 22 sampling stations were distributed across the lagoon (Figure 1) based on depth (<2, 2–4, and >4 m), sediment texture (sandy and muddy), and vegetation (bared, *Caulerpa* and *Cymodocea*) according to the information from previous studies (bathymetry from MAGRAMA, 2008; sediment characteristics from Álvarez-Rogel et al., 2019; macrophytes distribution from Belando et al., 2021). At each sampling point, in March and September of 2018, cold and warm season, respectively, and both characterized to be dry seasons in this area, a lineal transect of 25 m was fixed, depth and presence of vegetation was validated *in situ* by divers, and sediment samples were collected using cores (7-cm diameter) along the transect area as follows. Three surface sediment samples (4.5 cm) were collected for microbial analysis using cores (7 cm diameter) that were distributed at the beginning, center, and end of each transect. For physicochemical determination, five replicates per sampling point, collected every 5 m of the line transect, were homogenized prior to the analyses. The samples were not split by the depth due to the heterogeneous vertical profiles of physicochemical parameters in the first 4 cm among the samples from different stations, which hampered the definition of a vertical common cutoff for the oxic–anoxic boundary. The samples were taken to the laboratory in a portable 4°C cooler.

For microbial analyses, the individual sediment samples were weighted and manually homogenized after adding 50 ml of 1X PBS containing 3.5% NaCl. Then, for cell density measurements, 1 ml of each sample was fixed with formaldehyde (4% final concentration, 4 h at 4°C) and the fixation stopped with 9 ml of 1X PBS. Cell counts in each station were determined by epifluorescence microscopy after DAPI staining (Gomariz et al., 2015). The remaining homogenized sediments were stored at –80°C until processed for nucleic acid extraction (see below).

### Physicochemical measurements

Sediments for physicochemical analysis were processed immediately upon arrival at the laboratory. The redox potential (Eh) and pH values were measured three times per homogenized sample by inserting Eh and pH electrodes (Crison 50–55 and Crison 50–50, respectively). The Eh values were adjusted, according to Vepraskas and Faulkner (Vepraskas and Faulkner, 2001), by adding +200 mV to the measured voltage (the value of the Ag/AgCl reference electrode at 20°C). Then, sediment interstitial water was extracted by centrifugation at 11,300 *g* in a refrigerated centrifuge at 4°C under a nitrogen atmosphere. The dissolved sulfide was immediately measured (Cline, 1969). The rest of the interstitial water samples were stored at 4°C to measure electrical conductivity (EC), dissolved organic carbon (DOC), NO_3_^–^ and NH_4_^+^ within less than 1 week after the sampling. The DOC was measured in a TOC-automatic analyzer (TOC-VCSH Shimadzu). The NO_3_^–^ and NH_4_^+^ concentrations were measured with a V/UV spectrometer at λ = 220 nm and at λ = 670 nm, respectively, and the interference by organic matter was corrected by measuring the absorbance at λ = 275 nm (AOAC, 1975; Neiker, 2005). The solid material was dried at room temperature. Each dried sample was passed through a 2-mm mesh and a fraction larger than this size (mainly large mollusk shells) was discarded. The particle size was assessed with the 2-mm sieved aliquots, which were put in an ultrasonic bath for 10 min to promote particle dispersion. Then, they were sieved to 50 μm under a stream of water. Both fractions (sand, from 2 mm to 50 μm; silt + clay, <50 μm) were dried and weighed and the percentage of each one was calculated. Aliquots of the 2-mm sieved samples were crushed in an agate mortar for measuring total element concentrations (X-ray fluorescence). The amorphous or poorly crystalline Fe oxyhydroxides (FeA) were extracted by shaking 2-mm sieved samples for 6 h at 96°C with 30 ml 0.04 M hydroxylamine + 25% acetic acid (v/v) (Fortin et al., 1993). The degree of Fe pyritization (DOP) was calculated to establish the percentage of reactive iron incorporated into the pyrite fraction. The calculation was made using Eq. 1, assuming reactive-Fe to be extracted with dithionite (DOP-dithionite), according to the recommendations of Raiswell et al. (1994).


(1) $$\text{DOP}\left(\%\right)=\left(\frac{\mathrm{Pyrite}\mathrm{Fe}}{\mathrm{Pyrite}\mathrm{Fe}+\mathrm{Reactive}\mathrm{Fe}}\right)\times 100$$

The concentration of acid volatile sulfides (AVS = ΣFeS, Fe_3_S_4_, H_2_S) was determined in triplicate using 0.5–1.0 g of wet sample, according to the method described by Kostka and Luther (1994). Sulfide from AVS was liberated with 20 ml 3N HCl previously deaerated for 40–50 min. The sample was digested in a gas-tight reaction flask for 40–50 min under a continuous flow of nitrogen, which was bubbled through the flask as slowly as possible. The evolved H_2_S was then received in a trap containing 25 ml of 3% Zn acetate, 1 ml of concentrated H_2_SO_4_, and 4 ml of diamine reagent, and precipitated as ZnS. Sulfide was then measured colorimetrically with a UV-VIS spectrophotometer at a wavelength of 670 nm using the methylene blue method of Cline (Cline, 1969). Metal(loid)s simultaneously extracted with AVS (SEM-AVS), which is an approach to assess the potential for metal ions found in sediment to cause toxic effects in sediment-dwelling organisms, were analyzed by ICP-OES (Perkin Elmer, Optima model 4300 DV, Sunny vale, CA, United States) in 3N HCl.

### DNA extraction, 16S rRNA gene PCR amplification, and sequencing

Based on a 3D PCA with the physicochemical characteristics (Supplementary Figure 1), fourteen stations (corresponding to 82 samples, including triplicates per station and sampling time, except P8 with duplicates) were selected as representative of all the physicochemical and environmental conditions in the Mar Menor sediments and were analyzed microbiologically.

To extract DNA, the RNeasy Power Soil Total RNA Kit (Qiagen) and its complementary RNeasy Power Soil DNA Elution Kit (Qiagen) were used. Two grams of the homogenized sediment samples, stored at –80°C, were used according to the manufacturer’s instructions. Qubit™ dsDNA High Sensitivity (HS) or Broad Range (BR) (Invitrogen) kits were employed to measure DNA concentration.

The 82 extracted DNAs were used for PCR amplification of the 16S rRNA genes V3-V4 regions, using 25 cycles and primers 341F–805R (Takahashi et al., 2014). The PCR products from three independent reactions per sample were pooled and sequenced on an Illumina MiSeq using a 2 bp × 250 bp run (at Fundació per al Foment de la Investigació Sanitària i Biomédica, FISABIO, Valencia, Spain). Sequences were quality filtered removing sequences shorter than 50 bp or with a Phred quality below 30 by employing prinseq-lite (Schmieder and Edwards, 2011), and R1 and R2 reads were joined by FLASH (Magoč and Salzberg, 2011) using default parameters. Assembled sequences were analyzed using Qiime (Caporaso et al., 2010) for: (i) removing chimeras, using usearch6.1; (ii) clustering sequences in operational taxonomy units (OTUs) with 97% identity, using pick_open_reference_otus.py function; and (iii) assigning taxonomy of the longest sequence in each OTU, by BLAST against SILVA_132_release database at 97% identity. All samples were normalized at 47,461 sequences (the lowest number of sequences in a sample after post-assembly and filtering). To visualize the diversity and phylum or class abundances at each station and time of sampling, bubble plots were drawn in R with ggplot2 (Wickham, 2016).

### Statistical analysis

Due to the limitation of analyzing microbiologically the total amount of samples collected, and with the aim of selecting a set of stations representative of all the physicochemical and environmental conditions in the Mar Menor sediments, a principal component analysis (PCA) of the environmental variables was carried out using “prcomp.” The environmental data were normalized using the function “decostand” of the vegan package (Oksanen, 2011) with the parameter “range.” Correlations among the environmental variables were assessed by the function “cor” and variables with correlations coefficients above 0.85 were considered correlated.

Rarefactions curves were generated in R with the rarecurve function of the “vegan” package. The α-diversity measures (Shannon index and observed OTUs) were calculated with the “estimate_richness” function of the “phyloseq” package (McMurdie and Holmes, 2013). In addition, unique and shared OTUs among samples were displayed with the “UpSet” (visualizing intersecting sets) diagram using the “R- bioconductor” package “UpSetR” (Conway et al., 2017). For this analysis, we only considered OTUs above 0.1% in each sample which could be considered an abundant biosphere given the high diversity of these samples.

An analysis of variance (ANOVA) was used to test for statistical differences in physicochemical variables, concentration of cells, observed OTUs, Shannon index, and relative abundance of detected microbial classes among the levels of each of the following factors considered: Texture (levels: mud and sand), time (levels: March and September), vegetation (levels: bared, *Caulerpa* and *Cymodocea*), depth (levels: shallow, intermediate, and deep), and zone (levels: north, center, and south). Prior to ANOVA, homogeneity of variance was confirmed with Levene’s test (Levene, 1960). All *p*-values below 0.05 were considered significant.

To identify the main environmental factors that explain the changes in the microbial communities among the sampled stations, Bray–Curtis distances were calculated using the “vegdist” function of the “vegan” package, and a NMDS analysis was carried out. SIMPER analysis (Clarke, 1993) was used to identify species potentially responsible for statistically significant differences among levels of each factor using “vegan” (Oksanen, 2011).

Differences in the multivariate structure of microbial communities of each station among sampling times were tested using permutational multivariate analysis of variance (PERMANOVA) (Anderson, 2001; McArdle and Anderson, 2001) considering two factors (time and station). Wherein each term in the analysis was tested using 4,999 random permutations of the appropriate units and similarities among samples were calculated using the Bray–Curtis similarity index. Prior to PERMANOVA, homogeneity of multivariate dispersions among groups was tested with “betadisper.” All *p*-values below 0.05 were considered significant.

To get an explanation concerning the different behavior of the microbial communities between the two sampling times in the contaminated and non-contaminated stations, observed with the above PERMANOVA test, several statistical analyses were performed. First, contaminated and non-contaminated stations were defined by clustering the stations by FeA, PbAVS, and ZnAVS concentrations (indicators of metal pollution) using the function “agnes” and the ward method of the “cluster” R package (Supplementary Figure 2) (Maechler et al., 2019). Then, PERMANOVA with two factors: Time (levels: March–September) and PTEs [levels: contaminated (stations 4, 10–14) and non-contaminated (stations 1–3, 5 and 6)] was employed to test for differences in physicochemical parameters, following the same procedure explained above. Finally, to identify the variables responsible for the differences reported by PERMANOVA, a two-way ANOVA with the same factors was tested on each environmental variable. Additionally, using SIMPER analysis, metallophilic, intermediate, and non-metallophilic OTUs were defined based on the fold change among the mean abundance in the contaminated and non-contaminated stations. Metallophilic OTUs were defined as those having a fold change equal to or higher than 2, intermediate OTUs as those with fold change values higher than 0.5 but lower than 2, and non-metallophilic OTUs included those with a fold change lower than 0.5.

## Results and discussion

### General characteristics of the sediments

Based on a 3D PCA constructed with the physicochemical variables, 14 stations (among the 22 sampled stations; Supplementary Figure 1) that covered the variability of physicochemical conditions included in the plot, were selected as representative and analyzed more deeply. In these stations, redox potential (Eh) ranged from 197 to −240 mV with significantly lower values in muddy than sandy sediments (Supplementary Table 1.1 and Supplementary Figure 3). The Eh was significantly more negative in September than in March and sulfide (S^2–^) presented significantly higher concentrations in September, probably reflecting a higher activity of sulfate-reducing bacteria in the summer as observed in other systems (Robador et al., 2009). The pH values varied from nearly neutral to alkaline (pH: 7.1–8.4), with a tendency to lower values at muddy stations closer to the coast. Soluble organic carbon (SOC) was significantly higher in vegetated than in bare sediments with the highest values found in sediments colonized by *Cymodocea*. On the other hand, TOC displayed significantly higher concentrations in muddy than in sandy sediments, mainly in the north. Regarding DOP, which is related to sulfate reduction (Raiswell et al., 1988), statistically significant differences were observed in depth, with the higher values found in the deepest sediments, while NH_4_^+^, presented statistically significant differences between zones, with the higher values found in the south.

Acid volatile sulfide (AVS) was detected in very high concentrations in muddy sediments (≈2.8–141 mg kg^–1^), especially in the south of the lagoon, displaying statistically significant differences for both factors (texture and zone). High AVS concentrations in the Mar Menor bed had been previously reported (Marín-Guirao et al., 2005) and might be related to a high sulfate-reducing rate in the sediment favored by the high content of organic matter and the fine texture (Thode-Andersen and Jørgensen, 1989; Llobet-Brossa et al., 2002). Similarly, high concentrations of PTEs evaluated by means of FeAVS, ZnAVS, PbAVS, CdAVS, and AsAVS were found in almost all the muddy stations in both samplings, with high spatial variability (Supplementary Table 1.1). Particularly, stations located in the south of the lagoon (stations 10–14, see Figure 1), showed significant and unusually high concentrations of PbAVS (≈1,000–8,600 mg kg^–1^) and ZnAVS (≈600–3,900 mg kg^–1^). These results indicated metal(loid) pollution, mainly in the southern part of the lagoon, due to the runoff of mine wastes from the old mining area of Sierra de Cartagena-La Unión, in agreement with previously reported data (Marín-Guirao et al., 2008; María-Cervantes et al., 2009). Since PbAVS was correlated with CdAVS and FeA with FeAVS and ZnAVS, hereinafter we will only analyze PbAVS and FeA although their individual effects cannot be distinguished.

### Microbial abundances

**FIGURE 2:**
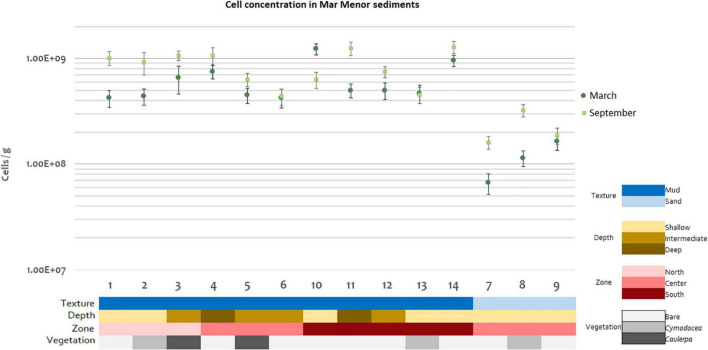
Microbial cell concentrations for each Mar Menor sediment station and time of sampling in logarithmic scale. One replicate per station and sampling time is shown. Error bars indicate standard deviation in DAPI count.

Cell concentrations ranged from 6.6 × 10^7^ to 130 × 10^7^ cells/g of sediment (Figure 2), values in the ranges previously reported for other marine sediments (Luna et al., 2002; Sørensen et al., 2007; García-Martínez et al., 2009). Statistically significant differences in cell concentrations were detected for texture (*p* = 0.000121), with higher concentrations in muddy than in sandy sediments (42 × 10^7^ to 130 × 10^7^ cells/g vs. 6.6 × 10^7^ to 32 × 10^7^ cells/g) and for zone (*p* = 0.0131), but not for time (*p* = 0.115), depth (*p* = 0.244), or vegetation (*p* = 0.457) (Supplementary Figure 4). Note that since the sandy samples were only present in the center of the lagoon, this zone presented lower cell concentrations than the north and the south. Despite no statistical differences being found for the factor time, it is noteworthy that microbial abundances were higher in September than in March for most of the studied stations (Figure 2), which is in agreement with previous studies (Musat et al., 2006; Suárez-Suárez et al., 2011).

### Overall microbial community description

The prokaryotic community of the Mar Menor sediments was analyzed by 16S rRNA gene metabarcoding. Although this is a powerful tool to investigate microbial community structure and dynamics, it has several limitations. For instance, biases due to different numbers of ribosomal RNA operons (Kembel et al., 2012), choice of amplification primers (Klindworth et al., 2013), and the amplification process (Suzuki et al., 1998; Sipos et al., 2007) have been reported. Furthermore, the bioinformatic pipeline is another source of bias, and Qiime1 is known to be very sensitive but usually overestimates diversity (Straub et al., 2020). Additionally, there is the possibility that some of these OTUs are derived from extracellular DNA from lysed cells (Torti et al., 2018). We are aware of these limitations and have tried to avoid overreaching conclusions.

**FIGURE 3:**
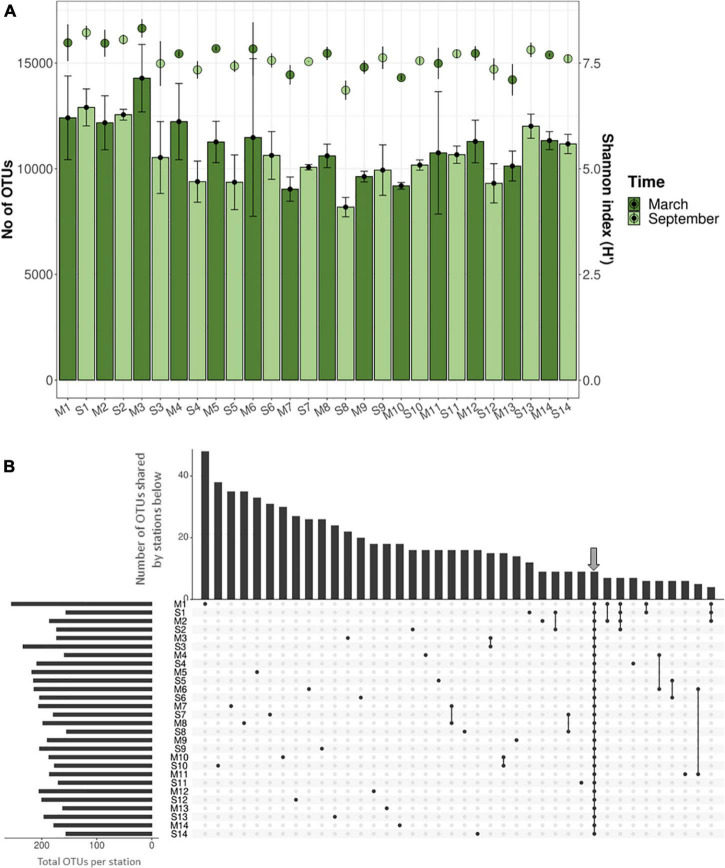
**(A)** Number of OTUs (columns) and Shannon index estimates (dots) for rarefied samples. Deviation bars indicate variability within replicates of each station and sampling time. **(B)** Upset plot showing the OTU distribution (for OTUs > 0.1% relative abundance) along the 14 stations during the cold and warm seasons (March and September, respectively). At the top, columns indicate the number of OTUs shared by stations, which are marked at the bottom with black dots. Arrow indicates the core microbiome.

For the microbiological analyses, DNA was extracted, amplified, and sequenced from 82 samples (including triplicates, except P8 with duplicates). A total of 3,891,802 reads were obtained and were grouped at 97% of similarity in 324,325 OTUs for the 82 samples after quality filtering, clustering, and normalizing (Supplementary Tables 1.2, 1.3). The rarefaction analysis indicated a good recovery of the microbial diversity inhabiting the sediments although the asymptote was not reached (Supplementary Figure 5). Regarding alpha diversity, the number of OTUs (richness) varied between 7,240 and 15,942, while the Shannon index values ranged from 6.7 to 8.5 (Figure 3A). The ANOVA test showed statistically significant differences in richness, and the Shannon index between textures (Shannon *p* = 0.009773; richness *p* = 0.001014) and zone (Shannon *p* = 2.695 × 10^–6^; richness *p* = 5.91 × 10^–6^), with higher values in muddy sediments and in the north area of the Mar Menor. However, similar values were found among times (Shannon *p* = 0.519; richness *p* = 0.122), depths (Shannon *p* = 0.567; richness *p* = 0.8468), or vegetations (Shannon *p* = 0.3696; richness *p* = 0.329) (Supplementary Figure 6).

The UpSet analysis, considering only OTUs with relative abundances above 0.1% [the “abundant” biosphere (Pedrós-Alió, 2012)], revealed that most of them were specific to individual stations with only a small proportion of shared OTUs (Figure 3B), highlighting the heterogeneity among stations. Nevertheless, a core microbiome of nine OTUs was detected in all stations and sampling times which represented a substantial fraction of the whole microbial community (from 2.4 up to 13.8% of total sequences) (Supplementary Table 1.4). The previous studies in intertidal sand and marine sediments (Sun et al., 2013; Boehm et al., 2014; Quero et al., 2015) have also detected both cosmopolitan (i.e., members of the core) and sample-specific microorganisms, likely representing generalists (i.e., members of the core) and specialists, respectively.

### Microbial community structure

**FIGURE 4:**
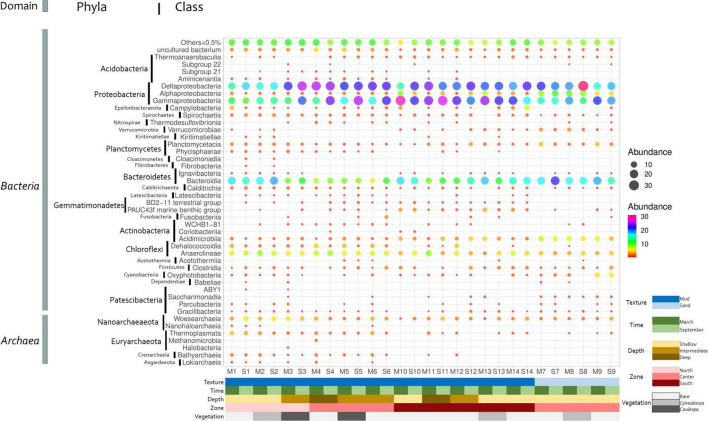
Distribution of bacterial and archaeal classes in Mar Menor sediments. Dot color and size indicate the relative abundance of each class. The main features of each station are shown at the bottom.

*Proteobacteria* was the phylum most frequently detected in all stations, accounting for 20.5–55.8% of the retrieved sequences (Supplementary Figure 7), followed by *Bacteroidetes* and *Chloroflexi* (4.3–26.4 and 1.9–17%, respectively). At the class level, *Deltaproteobacteria* (12.7–32.6%), now referred to as phylum *Desulfobacterota*, and *Gammaproteobacteria* (4.6–30.1%) dominated almost all the stations and times, with the exception of *Bacteroidia* dominating sample S7 and stations 1 and 2, both located on the channel connecting the lagoon with the Mediterranean Sea (Figure 4). The previous results on marine sediments from different locations also indicated the dominance of *Proteobacteria* and *Bacteroidetes* (Mahmoudi et al., 2015; Quero et al., 2015).

Although 16S rRNA gene metabarcoding provides little information regarding the metabolic capabilities of the community (Sun et al., 2020), some putative metabolisms can be hypothesized for some of the detected phyla in the Mar Menor sediments. For instance, the most frequently retrieved orders from the *Deltaproteobacteria* class were *Desulfobacterales* and *Desulfuromonadales*, which are typically composed of sulfate- and sulfur-reducing bacteria (Madigan et al., 2019). The order *Myxococcales* was also abundant in some samples, although very little is known about their physiology and metabolism in marine environments (Brinkhoff et al., 2012). This order has been associated with organic matter degradation in the seagrass rhizosphere (Ugarelli et al., 2017) and in *Posidonia oceanica* decaying material (Rubio-Portillo et al., 2021). In soil samples, members of *Myxococcales* present an aerobic metabolism, although some representatives are anaerobes that respire nitrate or Fe (III) (Treude et al., 2003) and produce a wide range of secondary metabolites (Reichenbach, 2015).

Within the *Gammaproteobacteria* class, the *Steroidobacterales* order was predominant in many stations, mainly due to the abundance of *Woeseia*, a genus with a versatile metabolism that goes from sulfur and hydrogen oxidation to chemorganoheterotrophy (Mußmann et al., 2017). The *Chromatiales* and *Ectothiorhodospirales* orders were also abundant. These groups grow autotrophically, acting as primary producers, using light and the energy provided by the oxidation of reduced sulfur compounds (Mori et al., 2015; Madigan et al., 2019; Sorokin et al., 2020).

The most frequently retrieved orders of the *Bacteroidia* class were *Flavobacteriales* and *Bacteroidales*, well-known degraders of polymeric organic matter (Fernández-Gómez et al., 2013; Bernardet, 2015; Krieg, 2015). Within this class, some OTUs could be assigned to genera within *Flavobacteriales*, which are aerobic or facultative aerobic bacteria with an obligate chemorganoheterotrophic metabolism (Cho and Giovannoni, 2004; Kim et al., 2008; Park et al., 2010; Choi et al., 2013; Bernardet, 2015). The OTUs assigned to *Bacteroidales* corresponded to uncultured genera, which hampers any metabolic inference, although most of the cultured members of this order are strict or facultative anaerobes (Krieg, 2015).

Considering only nine OTUs that formed the “abundant” core microbiome (Figure 3B and Supplementary Table 1.4), sulfate reduction and sulfur oxidation appeared also as potentially relevant and widespread metabolisms since three of these OTUs belonged to the *Desulfobacterales* and one to the *Chromatiales*. Furthermore, two OTUs related to *Bacteroidetes* were included in this core, highlighting the putative importance of polymeric organic matter degradation in the system.

In summary, based on the 16S rRNA gene diversity data, assuming all the limitations of this approach (Crosby and Criddle, 2003; Soergel et al., 2012; Ghyselinck et al., 2013; Rubio-Portillo et al., 2016) and the high proportion of OTUs related to uncultured microbes in the analyzed samples, the most frequently retrieved sequences corresponded to microorganisms related to S and C cycles, as expected for marine sediments (Plugge et al., 2011; Urich et al., 2014; Fike et al., 2015).

However, Mar Menor sediments also harbored a considerable degree of novelty since several uncultured groups were found in relatively high proportions. Sequences corresponding to the *Asgardeota* phylum accounted for up to 1.7% of the total sequences in some stations. Three classes of this phylum, *Lokiarchaeia*, *Heimdallarchaeia*, and *Odinarchaeia*, could be detected at relative abundances of 1.4, 0.3, and 0.2%, respectively. *Asgardarchaeota*, have been found in different aquatic sediments, and are known by their close phylogenetic relationship with *Eukarya*, thereby suggesting the emergence of eukaryotes within this phylum (Zaremba-Niedzwiedzka et al., 2017; Bulzu et al., 2019). The relative abundance of sequences related to *Heimdallarchaeia*, which seems to be the closest group to eukaryotes (Bulzu et al., 2019; Williams et al., 2020), is the highest reported so far.

Sequences corresponding to the *Woesearchaeia* class, within the phylum *Nanoarchaeota* and now classified as the order *Woesearchaeales*, were present in high proportions, accounting for up to 5.7% of the community. This group has been extensively detected around the world in many different ecosystems through 16S rRNA gene sequence studies, but little is known about their ecology and metabolism. The recent studies have shown the high diversity of this group and pointed to the oxic status as the main factor driving the distribution of Woesearchaeal members (Liu et al., 2018). A symbiotic or syntrophic lifestyle has been proposed although it has not been confirmed as a general trait of the group (Liu et al., 2018).

Finally, sequences from the *Actinobacteria* class WCHB1-81 accounted for up to 1.1% of the total. This group has been only described by 16S rRNA gene analyses so its metabolism remains completely unknown.

### Environmental factors driving microbial composition of sediment communities

**FIGURE 5:**
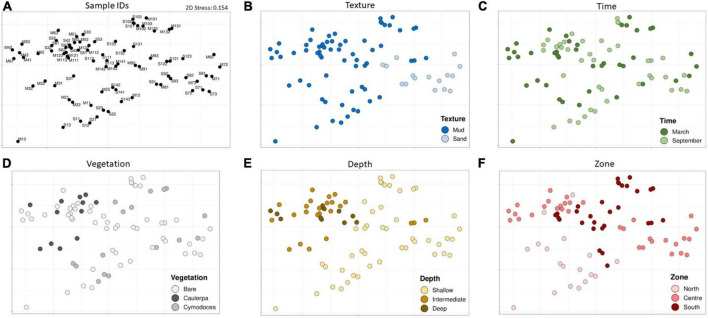
An NMDS plot of the Bray–Curtis distances among all samples **(A)**, colored by factor texture **(B)**, time **(C)**, vegetation **(D)**, depth **(E)**, and zone **(F)**.

To identify the main environmental factors related with the changes in the microbial communities, a NMDS plot based on Bray–Curtis distances of the microbial composition and abundance was performed. As shown in Figure 5, the texture (mud vs. sand), the type of vegetation (*Caulerpa* vs. *Cymodocea*), the depth (shallow vs. intermediate/deep), and the zone (north vs south) clustered the samples while factor time did not.

**FIGURE 6:**
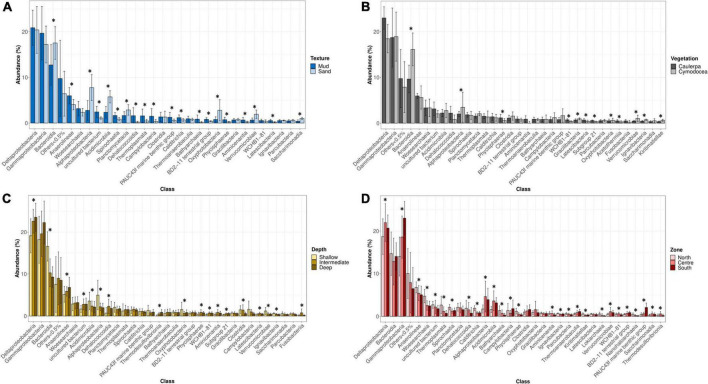
Relative abundance of detected microbial classes based on the levels of factor texture **(A)**, vegetation **(B)**, depth **(C)**, and zone **(D)**. Error bars indicate the range of abundances within each group of samples. Mud (*n* = 66); Sand (*n* = 16); *Cymo*docea *nodosa* (*n* = 16); *C. prolifera* (*n* = 16); Shallow (*n* = 46); Intermediate (*n* = 24); Deep (*n* = 12); North (*n* = 18); Centre (*n* = 34); and South (*n* = 30). Asterisk (*) indicates statistically significant differences tested by ANOVA (*p* < 0.05).

Texture is a well-known factor influencing the microbial community composition (Llobet-Brossa et al., 1998; Sun et al., 2013) and variables such as Eh, AVS, TOC, AsAVS, and FeA, which were statistically significant between levels of this factor (Supplementary Figure 3), could be the responsible of this differences. Although the influence of texture was expected, information about the relationship between sediment texture and microbial community structure still remains scarce in the literature (Boey et al., 2022). Thus, to provide some light, a detailed analysis of the taxonomic differences was performed. At the class level, a significant higher relative abundance of *Anaerolineae* was observed in muddy sediments while sequences related to *Bacteroidia*, *Alphaproteobacteria*, and *Acidimicrobiia* were significantly more abundant in sandy ones (Figure 6A). The most relevant OTUs highlighting the differences between muddy and sandy sediments were identified by SIMPER (Supplementary Table 1.5). Since microorganisms within the class *Anaerolineae* are strict anaerobes (Yamada and Sekiguchi, 2015), their relatively higher abundances in muddy sediments with a low redox potential were not surprising. On the other hand, sandy sediments showed a more positive redox potential and a lower concentration of total organic carbon. Accordingly, they harbored higher abundances of putatively aerobic microorganisms, such as the genera *Robiginitalea* and *Ulvibacter* (from the *Bacteroidia* class) (Cho and Giovannoni, 2004; Choi et al., 2007; Kim et al., 2008), and members of *Alphaproteobacteria* and *Acidimicrobiia*, which have been detected in aerobic and oligotrophic environments (Oh et al., 2012, 2016; Mizuno et al., 2015). In summary, these results suggested that sandy sediments had higher abundances of putatively aerobic microorganisms whereas muddy sediments had higher abundances of putatively anaerobic microbes.

Regarding the type of benthic vegetation (i.e., *Caulerpa* and/or *Cymodocea*), our result is in good agreement with previous comparisons between seagrasses and macroalgae (Gribben et al., 2017). Curiously, only SOC was statistically significant for this factor (Supplementary Figure 3). Given that *Caulerpa* was able to spread in the Mar Menor due to human activity, the taxonomic differences and their corresponding metabolic potentials are of great importance to determine the anthropogenic influence on the lagoon. At the class level, the proportion of sequences related to *Deltaproteobacteria* was higher in *Caulerpa*-associated sediments despite not being statistically significant, while *Bacteroidia* sequences had a statistically significant higher abundance in *Cymodocea*-associated sediments (Figure 6B). The OTUs contributing to the differences between *Caulerpa* and *Cymodocea* communities are shown in Supplementary Table 1.6. Our data agree with previous observations of higher abundances of sulfate-reducing microorganisms (*Deltaproteobacteria*) in *Caulerpa* sediments compared to phanerogam-colonized ones (Holmer et al., 2009; Aires et al., 2013; Gribben et al., 2017). Besides the difference in their relative proportions, different families of sulfate reducers were also present in both sediments (*Desulfobulbaceae* and *Desulfobacteraceae* in *Cymodocea* and *Caulerpa*-colonized sediments, respectively). *Desulfobacteraceae* are considered complete oxidizers while *Desulfobulbaceae* are unable to oxidize acetate to CO_2_ (Xing et al., 2020). On the other hand, different sulfur-oxidizing bacteria were also found associated with both types of vegetation, with B2M28, *Thioalkalivibrio* and *Thiohalophilus* dominating *Cymodocea* sediments and *Woeseia and Arenicellaceae* prevailing in *Caulerpa s*ediments. The class *Bacteroidia*, which was relatively more abundant in *Cymodocea* colonized sediments, has been found to be abundant in other seagrass species such as *Zostera marina*, *Z. noltii*, *Thalassia testudinum*, *and Syringodium filliforme*, with some phylotypes belonging to their core microbiome (Cúcio et al., 2016; Ugarelli et al., 2019). Furthermore, a study reported an enrichment in *Bacteroidales* inside a patch of the seagrass *Zostera* compared to the surrounding sediment (Ettinger et al., 2017). These observations suggest that some kind of stable association, direct or indirect, could exist between *Bacteroidia* and seagrasses, and is probably related to the higher content of labile organic matter inside seagrass patches compared to bare sediments (Honkoop et al., 2008; Ricart et al., 2015).

On the other hand, differences were also observed between the shallowest sediments (0–2 m) and those deeper than 2 m. This finding suggests that physicochemical conditions change at 2-m depth, shifting microbial communities, although only DOP, which was higher in deep sediments, presented statistically significant differences (Supplementary Figure 3). Intriguingly, a 2-m depth was also the limit for the survival of vegetation due to anoxia and light scarcity during the 2016 algal bloom (Belando et al., 2017). Regarding the taxonomic differences (Figure 6C), *Deltaproteobacteria* and *Anaerolineae*, both well-known anaerobic bacteria were significantly more abundant in intermediate/deep sediments while *Bacteroidia*, *Acidimicrobiia*, and *Alphaproteobacteria* were in shallow samples. The SIMPER analysis revealed the OTUs responsible for the differences between shallow and intermediate/deep samples (Supplementary Table 1.7). Remarkably, *Desulfobacteraceae* were enriched in intermediate/deep samples while *Robiginitalea* and *Actinomarinales* were in shallow samples. These differences are similar to those obtained with texture and based on the putative metabolic potential of these groups are probably due to oxygen availability.

Finally, we explored whether the difference between the north and the south microbial communities could be related to the extremely high concentrations of PTEs found in the southern basin (Supplementary Figure 3).

### Relationships between potentially toxic elements concentrations and microbial communities

As explained above, clear differences in the microbial communities were observed between zones, mainly among the north and south microbial communities (Figure 5E), most likely due to the extremely high concentrations of PTEs present in the southern basin attributable to the presence of mining wastes carried from the old mining area (Marín-Guirao et al., 2008; María-Cervantes et al., 2009) (Supplementary Figure 3). Remarkably, when the temporal changes in the microbial communities of each station were tested by PERMANOVA (two factors: station and time) significant *p*-values were obtained (*p* = 0.0002), and *a posteriori* test indicated that the stations with the highest PTEs concentrations presented significant temporal changes while the less contaminated stations did not (Table 1). These differences were mainly due to changes in low abundance bacterial classes but some relatively abundant ones (up to 6.2% relative abundance) also displayed extensive changes. These differences between the most contaminated (P4, P10, P11, P12, P13, and P14) and the less contaminated stations (which we will refer to as contaminated and non-contaminated, respectively) can be explained by the following three hypotheses: (i) Contaminated and non-contaminated stations were exposed to different temporal environmental changes, (ii) there was an input of PTEs in the contaminated stations between March and September, and (iii) microbial communities inhabiting the sediments with high PTEs concentrations are more vulnerable to environmental changes. The PERMANOVA test of the environmental variables with two factors (time: March vs. September; PTEs: contaminated vs. non-contaminated), indicated that there were significant differences in the whole lagoon between March and September and between the contaminated and non-contaminated stations but not in the interaction between the two factors (Supplementary Table 1.8) which means that there was no evidence that temporal changes among the two groups of stations were different and thus, “hypothesis i” was rejected. Then, the two-way (time and PTEs) ANOVAs (Supplementary Table 1.9) identified redox potential and S^2–^ as the variables responsible for temporal changes, and AVS and PTEs, for the differences between contaminated and non-contaminated stations. Thus, there was no evidence of input of PTEs between March and September and therefore “hypothesis ii” was also rejected. The only environmental variables that differed between contaminated and non-contaminated stations were those associated with PTEs. Therefore, based on our results, the most likely “hypothesis iii” is that the microbial communities that inhabit the sediments with high PTEs concentrations are more vulnerable to environmental changes. Further, *in vitro* experiments would be necessary to confirm the hypothesis.

**TABLE 1:** The *p*-values of the PERMANOVA *a posteriori* test for the microbial communities of each station between March and September.

	PTEs	*p* (MC)
M1-S1	Non-contaminated	0.1678
M2-S2	Non-contaminated	0.0724
M3-S3	Non-contaminated	0.0976
M4-S4	Contaminated	0.0350*
M5-S5	Non-contaminated	0.1974
M6-S6	Non-contaminated	0.1154
M7-S7	Non-contaminated	0.0112*
M8-S8	Non-contaminated	0.1802
M9-S9	Non-contaminated	0.0472*
M10-S10	Contaminated	0.0178*
M11-S11	Contaminated	0.0142*
M12-S12	Contaminated	0.0084*
M13-S13	Contaminated	0.0454*
M14-S14	Contaminated	0.0268*

MC, Monte Carlo correction. *p < 0.05.

Microbes able to survive in the presence of high concentrations of PTEs (heavy metals and As), which can be considered an extreme condition, are known as metallophiles (Nies, 2000; Gupta et al., 2014). SIMPER analyses supported our hypothesis by revealing that temporal changes in PTE-contaminated samples were mainly due to metallophilic OTUs (defined as those OTUs statistically different or contributing to changes among contaminated and non-contaminated stations and whose mean relative abundance in contaminated stations was two-fold higher than in non-contaminated stations) (19.64–95.15%) (Table 2). Metallophilic OTUs could correspond to specialist bacteria harboring mechanisms to resist or use the PTEs which could be more susceptible to environmental perturbations. Indeed, it has been previously reported that such specialist microorganisms can be more vulnerable to fluctuations in the other environmental variables since the genetic adaptation to one environment may result in loss of fitness in another environment (Cooper and Lenski, 2000; Kassen, 2002; Zhong et al., 2004). Nevertheless, non-metallophilic OTUs also contributed to temporal changes but in a lower proportion (5.44–22.07%).

**TABLE 2:** Percentage of OTUs classified as metallophilic (fold change > 2), intermediate (0.5 < fold change < 2), and non-metallophilic (fold change < 0.5) that were also statistically different in the PTE contaminated samples between March and September.

		Significant OTUs PTE samples vs. time (%)
Significant OTUs among PTE and non-PTE samples	Metallophilic (224)	19.64
	Intermediate (328)	17.68
	Non-metallophilic (1,765)	5.44

		**OTUs contributing to changes in PTE samples vs. time (%)**

OTUs contributing to changes among PTE and non-PTE samples	Metallophilic (1,588)	95.15
	Intermediate (3,182)	74.89
	Non-metallophilic (2,097)	22.07

Numbers in parenthesis indicate the total number of OTUs within each group.

Some of the extreme metallophilic OTUs found in the Mar Menor sediments have been previously associated with metal-rich environments. Metallophiles were identified by examining the Upset diagrams, which showed that P10, the most contaminated station, harbored a set of 15 specific OTUs that were present between March and September with abundances higher than 0.1% (Figure 3B). These OTUs were assigned to Sva1033, *Woeseiaceae*, *Anaerolineaceae*, OPB41, *Desulfobulbaceae*, *Desulfuromusa*, *Flavobacteriaceae*, and SAR324 families. Sva1033 metabolism has been associated with metal reduction (Sun et al., 2013; Buongiorno et al., 2019). *Woeseia* was the most abundant genus in a copper biofilm (Zhang et al., 2019) and metagenomic data suggested a broad range of metabolism for *Woeseiaceae*, including sulfur-based chemolithoautotrophy (Mußmann et al., 2017). OPB41 refers to a 16S rRNA gene amplicon sequence detected for the first time in a Yellowstone hot spring, an environment with high concentrations of reduced iron and sulfur (Hugenholtz et al., 1998). *Desulfobulbaceae* and SAR324 have been reported to negatively correlate with toxic elements (Baltar et al., 2018; Wu et al., 2019); however, the OTUs detected herein could be new representatives of these groups since their identities to sequences in the ARB-SILVA database were rather low (95.71 and 95.92%, respectively). *Flavobacteriaceae* was present in toxic metal-contaminated marine sediment (Gillan et al., 2005; Toes et al., 2008) and species of the genus *Desulfuromusa* are well-known Fe-reducing bacteria (Vandieken et al., 2006).

Remarkably, two out of the nine core OTUs (those shared between all stations and sampling times) with abundances >0.1% (NR_OTU1827 and AF286033.1.1521) were also defined as extreme metallophiles and belonged to the classes *Gammaproteobacteria* and PAUC43f (*Gemmatimonadota* phylum), respectively. As shown in Figure 6D, there were statistically significant higher abundances of *Gammaprotebacteria* and PAUC43f in the south of the lagoon, the most contaminated area. Sequences similar to this *Gammaproteobacteria* OTU have been detected in heavy metal-rich environments (Ben Said et al., 2010; Meyer-Dombard et al., 2012), a microbial mat at a shallow submarine hot spring (Hirayama et al., 2007), and marine sediments (Schöttner et al., 2011), as well as in the guts of fishes (NCBI accession number KJ197761). Regarding the PAUC43f 16S rRNA gene sequence (NCBI accession number AF286033), it was first detected in a saltmarsh contaminated with mercury and polychlorinated-biphenyls (PCBs). Closely related sequences were also detected in different types of sediments (Bowman and McCuaig, 2003; López-García et al., 2003; Genderjahn et al., 2018) and as a symbiont of marine invertebrates (Radwan et al., 2010; Griffiths et al., 2019; Bergo et al., 2021). Although it was deposited in the NCBI database more than 20 years ago and it has been found even in different ecosystems, there is no available information about the metabolism of this class. The high abundance and widespread presence of PAUC43f in the Mar Menor sediments make it an excellent scenario to study the characteristics of this uncultured class.

## Conclusion

The Mar Menor lagoon is subjected to natural and intense anthropic pressures, which have left their imprints on the microbial community inhabiting the sediments. Here, we have shown that texture, type of vegetation, depth, and zone are strong structuring factors while the effect of time (season) is more limited and restricted to PTE contaminated stations. Our results provide a detailed description of the differences in microbial composition associated with these factors which are of great importance for the health of the lagoon. For instance, *Caulerpa* colonized sediments harbor a higher potential for sulfate reduction and thus a lower redox potential (as we have observed in aquaria experiments, data not shown). This human-introduced factor in the Mar Menor can be crucial in determining the response of the sediment microbiota to changes in the water column, which may favor the proliferation of sulfide-producing microbes that negatively affect the system. However, the sediment microbial community also harbors the potential for detoxification as well as microbes that are interesting both because of their novelty and resistance to PTEs. The history of mining impacts has most likely influenced the microbial communities, which have developed ways to tolerate, and even thrive, in this highly polluted surrounding. However, this specialization may have also increased the vulnerability of these communities to other environmental changes which add more instability to the whole ecosystem.

The sediment microbiota could be a key player in the fate of the Mar Menor since they could buffer the system from, and respond to, environmental changes. Here, we paved the way to understanding how this may occur.

## Data availability statement

16S rRNA gene sequences generated in this work have been deposited in the NCBI Sequence Read Archive (SRA) database under BioProject PRJNA753791.

## Author contributions

BA-R: conceptualization, data curation, formal analysis, investigation, methodology, validation, visualization, writing—original draft, and writing—review and editing. ER-P: data curation, formal analysis, investigation, methodology, supervision, validation, visualization, writing—original draft, and writing—review and editing. JÁ-R: conceptualization and writing—review and editing. FG-C: conceptualization, methodology, visualization, and writing—review and editing. XO: conceptualization, data curation, formal analysis, investigation, methodology, supervision, validation, visualization, writing—original draft, and writing—review and editing. M-DB: data curation and writing—review and editing. JB-E: conceptualization, data curation, funding acquisition, methodology, project administration, resources, and writing—review and editing. RG-M: data curation, project administration, and writing—review and editing. AF: data curation, formal analysis, methodology, supervision, writing—original draft, and writing—review and editing. JR: conceptualization, funding acquisition, methodology, project administration, resources, and writing—review and editing. FS: conceptualization, investigation, supervision, validation, visualization, writing—original draft, and writing—review and editing. JA: conceptualization, funding acquisition, investigation, project administration, supervision, validation, visualization, writing—original draft, and writing—review and editing. All authors contributed to the article and approved the submitted version.
