# Supplementary material for: Factors structuring microbial communities in highly impacted coastal marine sediments (Mar Menor lagoon, SE Spain)
Source: Front Microbiol. 2022 Sep 7;13:937683. doi: 10.3389/fmicb.2022.937683 (PMC9491240; doi:10.3389/fmicb.2022.937683)
Supplement: Supplementary file 1 [file Data_Sheet_1.docx]

Supplementary Material

**Factors structuring microbial communities in highly impacted coastal marine sediments (Mar Menor lagoon, SE Spain)**

Borja Aldeguer-Riquelme^1^, Esther Rubio-Portillo^1^, José Álvarez-Rogel^2^, Francisca Giménez-Casalduero^3^, Xose Luis Otero^4^, María-Dolores Belando^5^, Jaime Bernardeau-Esteller^5^, Rocío García-Muñoz^5^, Aitor Forcada^3^, Juan M. Ruiz^5^, Fernando Santos^1^, Josefa Antón^1,6*^

^1^ Department of Physiology, Genetics, and Microbiology, University of Alicante, 03080 Alicante, Spain

^2^Department of Agricultural Engineering of the ETSIA & Soil Ecology and Biotechnology Unit of the Institute of Plant Biotechnology, Technical University of Cartagena, 30203 Cartagena, Spain

^3^Department of Marine Science and Applied Biology, University of Alicante, 03080 Alicante, Spain

^4^CRETUS, Departamento de Edafoloxía e Química Agrícola, Facultade de Bioloxía, Universidade de Santiago de Compostela, Spain

^5^Seagrass Ecology Group, Spanish Oceanography Institute of the Spanish National Research Council, Oceanography Center of Murcia. C/Varadero 1, 30740 San Pedro del Pinatar, Murcia, Spain

^6^Multidisciplinary Institute of Environmental Studies Ramón Margalef, University of Alicante, 03080 Alicante, Spain

*Correspondence:

Josefa Antón

E-mail: [anton@ua.es](mailto:anton@ua.es)

Tel. +34 965 90 38 70, Fax. +34 965 90 95 69

***
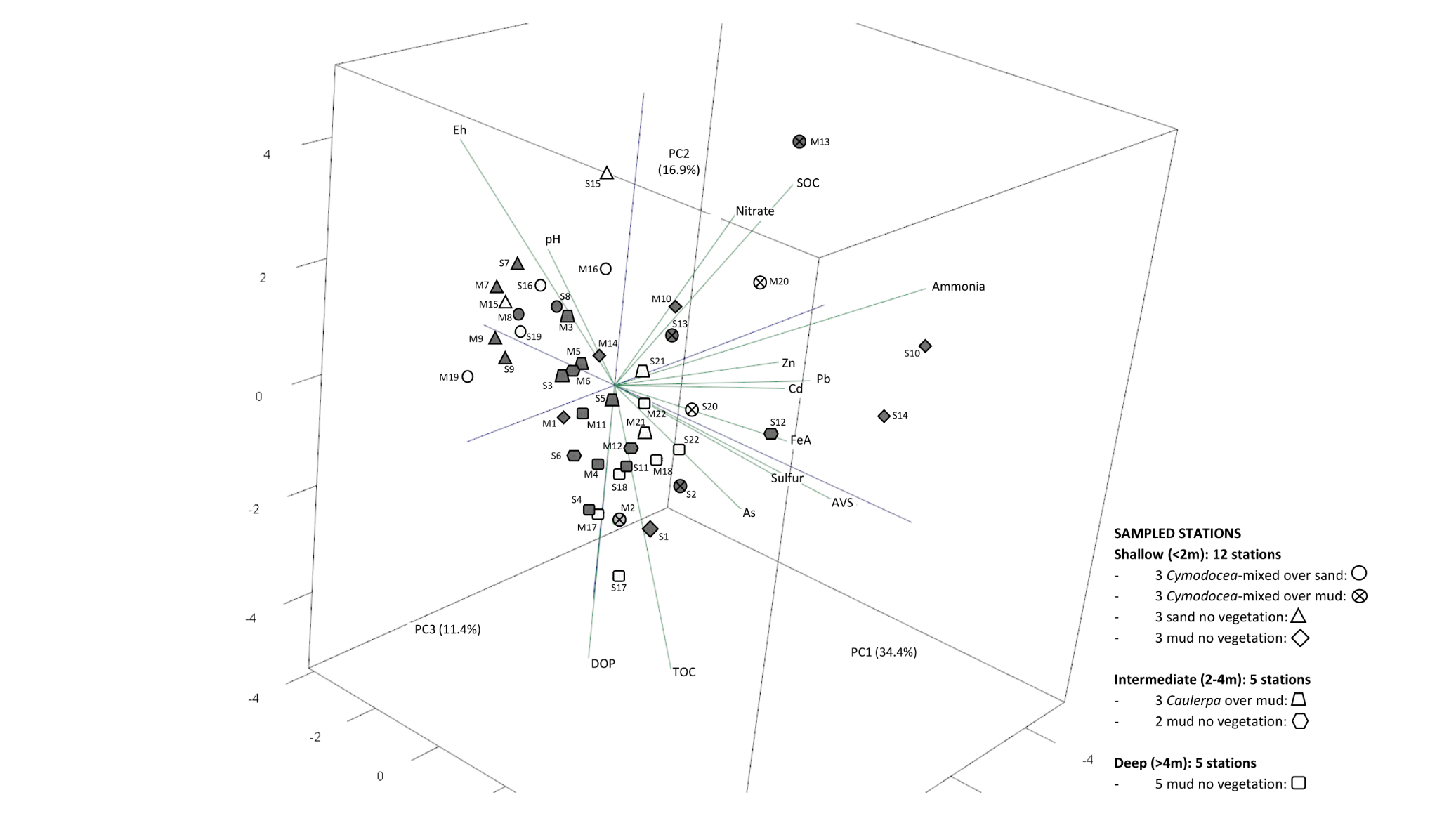
1.Supplementary* *figures***

**Suppl. Fig. S1.** Principal component analysis of environmental variables of the 22 sampled stations in March and September 2018. Different symbols indicate sampling depth (< 2 m, 2-4 m, and >4 m), sediment texture (sand and mud) and vegetation (bare, *Caulerpa* and *Cymodocea*) and those symbols colored in grey indicate stations selected for microbiological analyses, following the same color code as in figure 1.


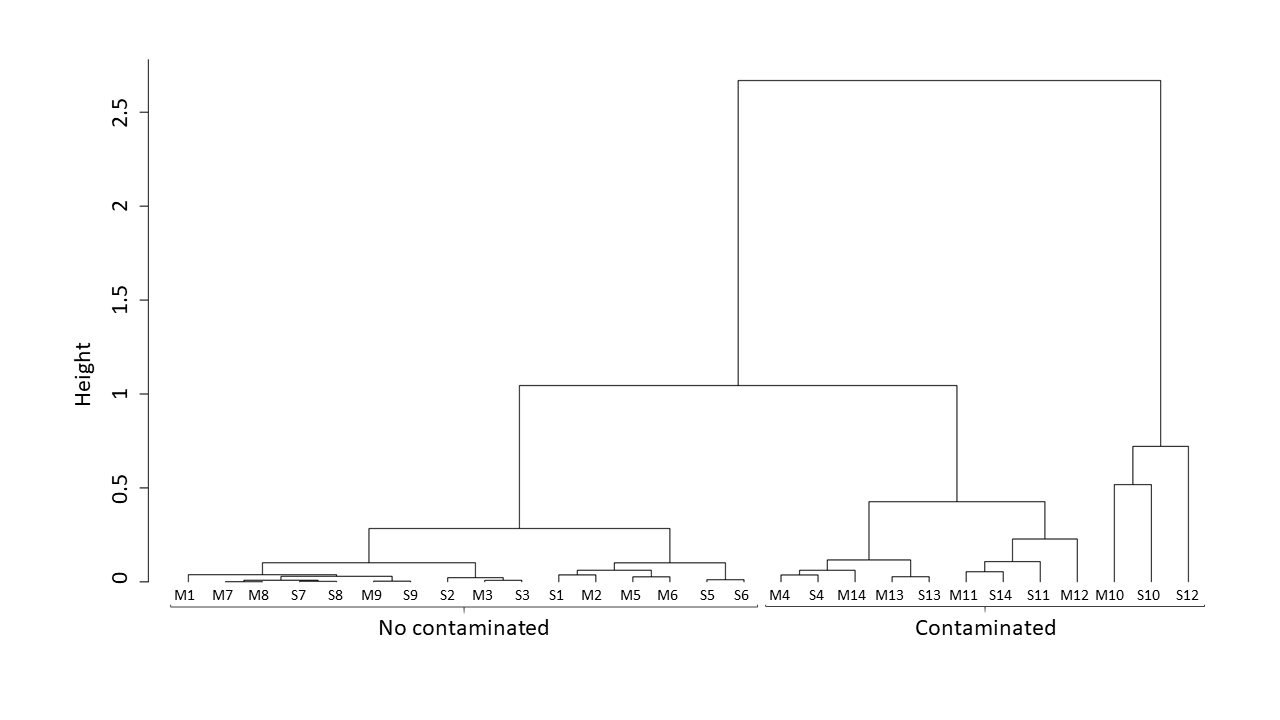


**Suppl. Fig. S2.** Sample clustering based on FeA, PbAVS and ZnAVS concentrations, indicators of mining pollution.


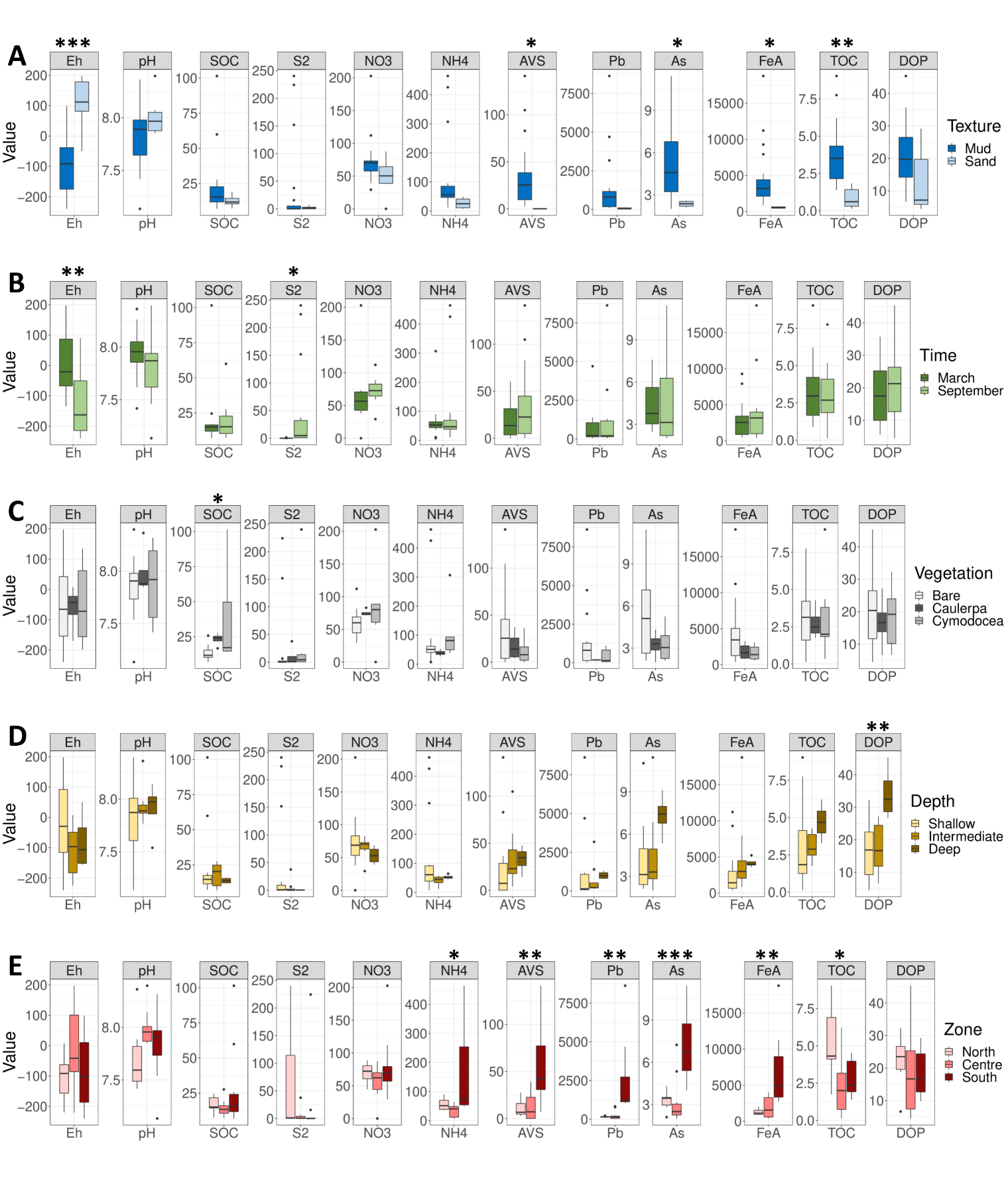


**Suppl. Fig. S3.** Boxplots of the physicochemical variables for levels of the factors texture (A), time (B), vegetation (C), depth (D) and zone (E). Second and third quartile (25-75%) are included in the box while the first (0-25%) and fourth quartile (75-100%) are represented by lines below and above the box, respectively. The line inside the box indicate the mean and the black dots represent outliers. Asterisks (*) indicate statistically significant differences tested by ANOVA (*: p-value<0.05; **: p-value<0.01; ***: p-value<0.001).


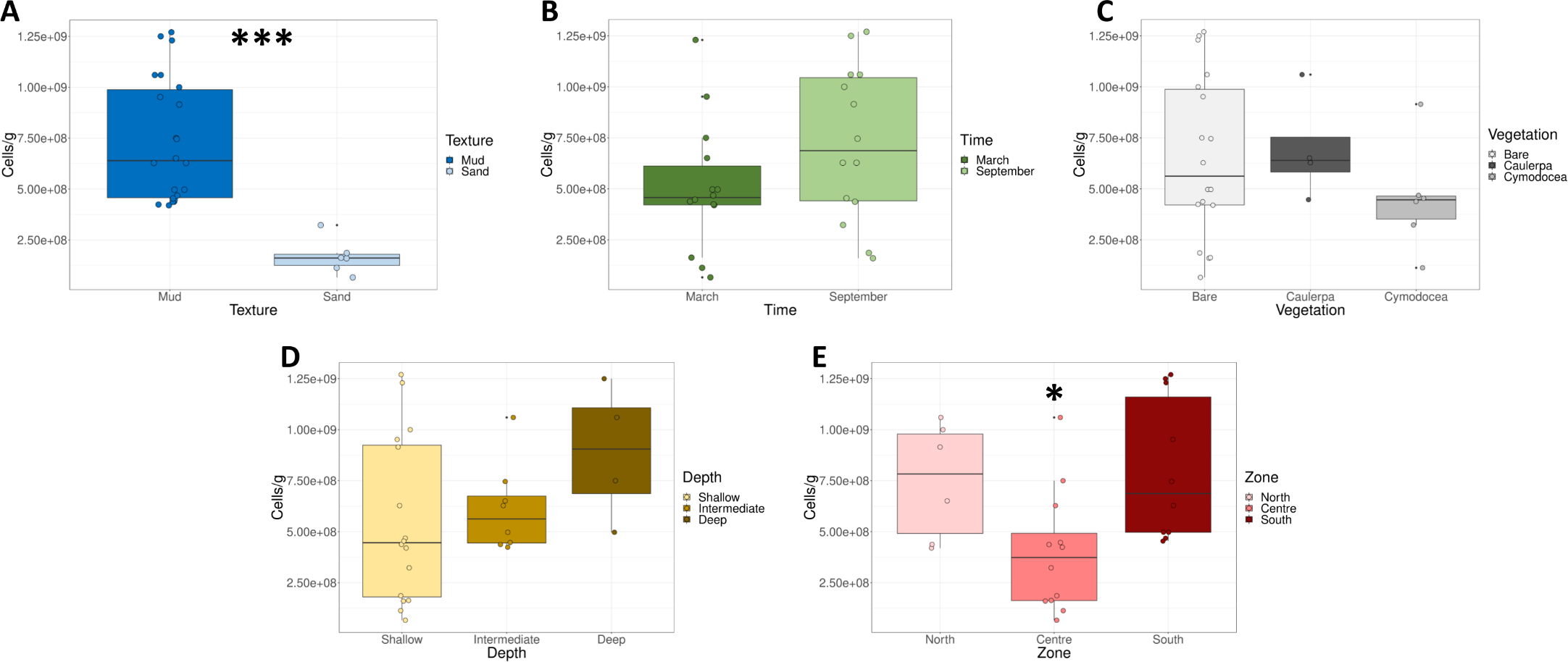
**Suppl. Fig. S4.** Boxplots of the concentration of cells for levels of the factor texture (A), time (B), vegetation (C), depth (D) and zone (E). Second and third quartile (25-75%) are included in the box while the first (0-25%) and fourth quartile (75-100%) are represented by lines below and above the box, respectively. The line inside the box indicate the mean and the black dots represent outliers. Asterisks indicate statistically significant differences tested by ANOVA (*: p-value<0.05; ***: p-value<0.001).


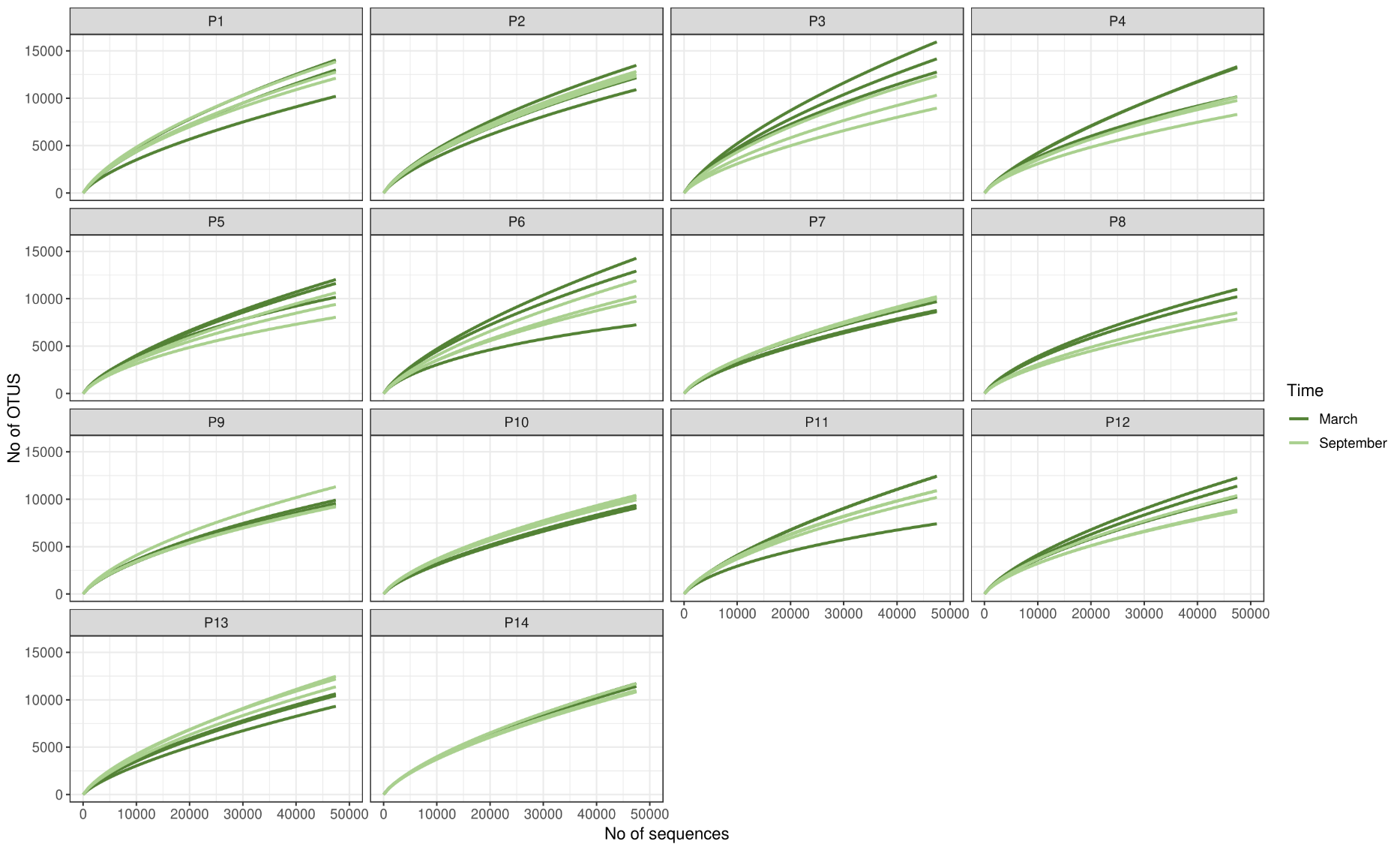


**Suppl. Fig. S5.** Rarefaction curves of the 16S rRNA gene sequences obtained for each sample. Lines are colored by the time of sampling.


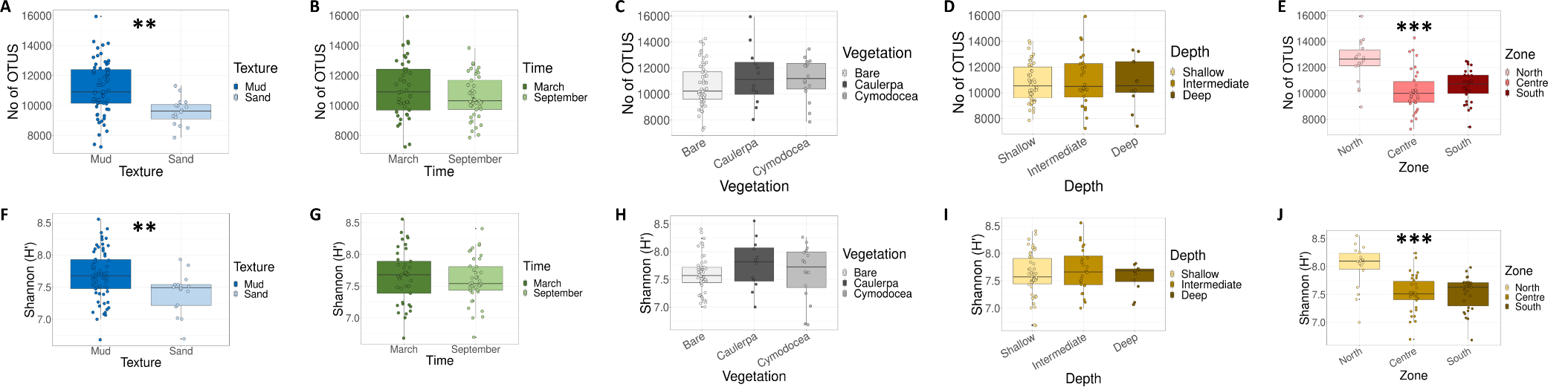


**Suppl. Fig. S6.** Boxplot of observed OTUs (A-E) and Shannon index (F-J) for levels of the factor texture (A and F), time (B and G), vegetation (C and H), depth (D and I) and zone (E and J). Second and third quartile (25-75%) are included in the box while the first (0-25%) and fourth quartile (75-100%) are represented by lines below and above the box, respectively. The line inside the box indicates the mean and the black dots represent outliers. Asterisks (*) indicate statistically significant differences tested by ANOVA (**: p-value<0.01; ***: p-value<0.001).


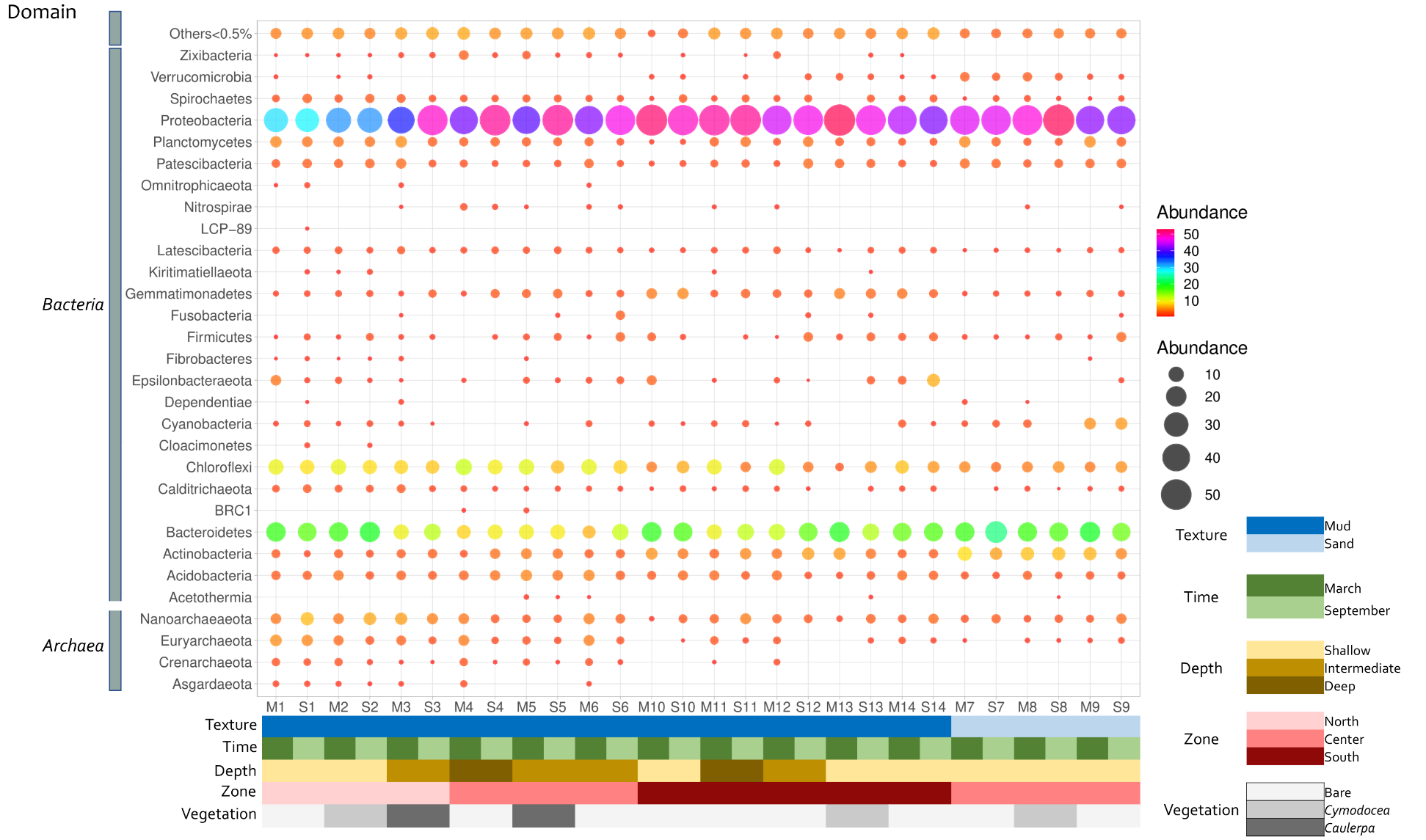
**Suppl. Fig. S7.** Taxonomic distribution of Mar Menor sediments at the phylum level. Color and size of dots indicates the relative abundance of each class. The main features of each station are shown at the bottom.

***2. Supplementary tables***

**Suppl. Table 1. Supplementary Materials. S1.1.** Physicochemical data of Mar Menor sediments. TOC: total organic carbon; DOP: degree of pyritization; DOC: dissolved organic carbon; Element AVS: PTE simultaneously extracted with acid volatile sulfides (AVS); FeA: amorphous Fe oxyhydroxides. b.d.l.: below detection limits. **Table S1.2.** General characteristics of libraries employed for the analysis of microbial communities and alpha diversity data. **Table S1.3.** Number of reads belonging to each OTU (rows) in each sample (columns). Data is rarefied to 47461 reads per sample. ANOVA test of the observed OTUs and Shannon index, with the factors time (March/September) and texture (mud/sand). **S1.4.** Relative abundances of the nine OTUs detected in all stations and sampling times representing more than 0.1%. Each sample site and time is represented by a column. **S1.5.** SIMPER analysis of the OTUs present in microbial communities from muddy versus sandy sediments. OTUs are ordered by percentage of contribution to dissimilarities. **S1.6.** SIMPER analysis of the OTUs present in microbial communities associated with *Caulerpa prolifera* versus *Cymodocea nodosa* sediments. **S1.7.** SIMPER analysis of the OTUs present in microbial communities associated with shallow versus intermediate and deep sediments. **S1.8.** PERMANOVA analysis for the physicochemical parameters using factor “Time” with two levels (March and September) and “PTEs” with two levels [Contaminated (P4, P10, P11, P12, P13 and P14) and non-contaminated (P1, P2, P3, P5 and P6)]. **S1.9.** Two-way ANOVA test for each physicochemical variable with factor “Time” with two levels (March and September) and “PTEs” with two levels Contaminated (P4, P10, P11, P12, P13 and P14) and non-Contaminated (P1, P2, P3, P5 and P6)]. P-values lower than 0.05 are shown in grey.

The supplementary tables can be found in the following link:
<https://mega.nz/folder/JJNxgQwK#AmrrCxnUCYwFfilCdf9FnA>
